# Protein Phosphatase 2A Controls Ethylene Biosynthesis by Differentially Regulating the Turnover of ACC Synthase Isoforms

**DOI:** 10.1371/journal.pgen.1001370

**Published:** 2011-04-21

**Authors:** Kyle R. Skottke, Gyeong Mee Yoon, Joseph J. Kieber, Alison DeLong

**Affiliations:** 1 Department of Molecular Biology, Cell Biology, and Biochemistry, Brown University, Providence, Rhode Island, United States of America; 2 Department of Biology, The University of North Carolina at Chapel Hill, Chapel Hill, North Carolina, United States of America; The Salk Institute for Biological Studies, United States of America

## Abstract

The gaseous hormone ethylene is one of the master regulators of development and physiology throughout the plant life cycle. Ethylene biosynthesis is stringently regulated to permit maintenance of low levels during most phases of vegetative growth but to allow for rapid peaks of high production at developmental transitions and under stress conditions. In most tissues ethylene is a negative regulator of cell expansion, thus low basal levels of ethylene biosynthesis in dark-grown seedlings are critical for optimal cell expansion during early seedling development. The committed steps in ethylene biosynthesis are performed by the enzymes 1-aminocyclopropane 1-carboxylate synthase (ACS) and 1-aminocyclopropane 1-carboxylate oxidase (ACO). The abundance of different ACS enzymes is tightly regulated both by transcriptional control and by post-translational modifications and proteasome-mediated degradation. Here we show that specific ACS isozymes are targets for regulation by protein phosphatase 2A (PP2A) during *Arabidopsis thaliana* seedling growth and that reduced PP2A function causes increased ACS activity in the *roots curl in 1-N-naphthylphthalamic acid 1* (*rcn1)* mutant. Genetic analysis reveals that ethylene overproduction in PP2A-deficient plants requires *ACS2* and *ACS6*, genes that encode ACS proteins known to be stabilized by phosphorylation, and proteolytic turnover of the ACS6 protein is retarded when PP2A activity is reduced. We find that PP2A and ACS6 proteins associate in seedlings and that RCN1-containing PP2A complexes specifically dephosphorylate a C-terminal ACS6 phosphopeptide. These results suggest that PP2A-dependent destabilization requires RCN1-dependent dephosphorylation of the ACS6 C-terminus. Surprisingly, *rcn1* plants exhibit decreased accumulation of the ACS5 protein, suggesting that a regulatory phosphorylation event leads to ACS5 destabilization. Our data provide new insight into the circuitry that ensures dynamic control of ethylene synthesis during plant development, showing that PP2A mediates a finely tuned regulation of overall ethylene production by differentially affecting the stability of specific classes of ACS enzymes.

## Author Summary

Like animals, plants produce a number of substances that regulate growth and coordinate developmental transitions and responses to environmental signals. Ethylene gas is one such regulator of the plant life cycle, playing important roles in fruit ripening, pathogen defenses, and the regulation of cell expansion. Because overall plant form is determined largely by the degree and directionality of cell expansion, ethylene is a crucial regulator of morphology, and ethylene production must be maintained at low levels during phases of rapid cell expansion, such as early seedling growth. Recent work has identified molecular mechanisms that target ethylene biosynthetic enzymes for proteolytic degradation; this degradation plays a key role in controlling ethylene production. Here we exploit the molecular genetic resources available in the *Arabidopsis thaliana* system to identify a highly conserved protein complex that dephosphorylates target proteins as a new component of the mechanism that regulates degradation of ethylene-producing enzymes. Our findings show that protein phosphatase 2A plays a nuanced role in this regulatory circuit, with both positive and negative inputs into the stability of specific proteins that drive ethylene biosynthesis. This work enhances our understanding of the mechanisms that enforce adaptive levels of hormone production in plants.

## Introduction

Ethylene gas is a crucial regulator of numerous aspects of plant development and physiology, including germination, seedling growth and morphology, organ senescence and fruit ripening, as well as stress and defense responses [1]. The biosynthetic capacity for ethylene production is nearly ubiquitous throughout the plant body, but biosynthesis is generally maintained at low levels through regulatory circuitry that confers tight control while allowing rapid and dramatic increases under conditions such as wounding or fruit ripening. Ethylene biosynthesis levels change in response to endogenous developmental cues as well as light, temperature, pathogens and other exogenous signals (reviewed in references [2]–[4]).

**Figure 1:**
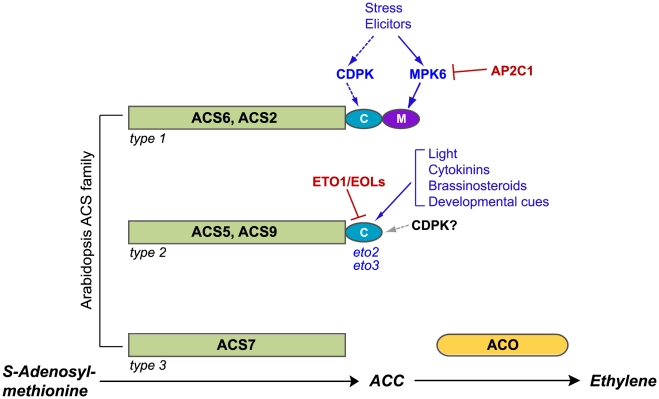
Post-translational modifications of ACS proteins regulate ethylene synthesis. The three ACS isozyme types are defined by their C-terminal amino acid sequence motifs; representative members of each type are shown. Consensus sites for MAPK and CDPK phosphorylation are shown as purple and turquoise ovals, respectively. Several factors and signals that affect the stability of type 1 and type 2 isozymes through action on C-terminal motifs are indicated, with positive regulators shown in blue and negative regulators shown in red. In the absence of a stabilizing input, type 1 and type 2 isozymes are rapidly degraded by the 26S proteasome. Evidence for CDPK regulation of type 1 isozymes has been obtained in tomato fruits; dashed lines indicate that this role has not yet been established in Arabidopsis. Although the C-terminus of type 2 isozymes can be phosphorylated by CDPKs in vitro, a regulatory role for CDPK phosphorylation of these proteins has not been demonstrated in vivo; the gray arrow indicates possible CDPK input. The *eto2* and *eto3* mutations are stabilizing alleles of ACS5 and ACS9, respectively. Not depicted in this cartoon are ACS4 and ACS8, two additional type 2 isozymes, and ACS1, a catalytically inactive type 1 isozyme.

Ethylene is derived from methionine via a well-characterized biosynthetic pathway (reviewed in references [4]–[7]) in which the first committed step, conversion of *S*-adenosyl methionine to 1-aminocyclopropane 1-carboxylate (ACC), is performed by the enzyme ACC synthase (ACS; see Figure 1). The final step is the conversion of ACC to ethylene, CO_2_ and cyanide by ACC oxidase (ACO). Under some conditions (particularly in fruit and flowers), ACO activity may be rate limiting, but ACC synthesis is generally the rate-limiting step for ethylene production during vegetative growth. In seedlings, increasing ACS protein levels drive ethylene synthesis to high levels [8]–[10].

ACS isozymes are encoded by a gene family comprising three subclasses defined by the absence or presence of C-terminal phosphorylation motifs (Figure 1). Type 1 ACS isozymes carry target sites for mitogen-activated protein kinase (MAPK) phosphorylation; this MAPK motif lies immediately downstream from a calcium-dependent protein kinase (CDPK) phosphorylation site [11]–[13]. Type 2 isozymes carry only the CDPK target motif, and type 3 isozymes carry neither target site. Phosphorylation of the type 1 isozymes ACS2 and ACS6 by stress-responsive MAPKs (MPK3 and MPK6) results in increased ethylene synthesis through protein stabilization [10], [13], [14]. Unphosphorylated type 1 isozymes are rapidly turned over via a 26S proteasome-dependent pathway, and the non-catalytic C-terminal region containing the CDPK and MAPK phosphorylation motifs is sufficient to confer instability on reporter protein fusions [10]. CDPK-mediated phosphorylation of LeACS2, a tomato type 1 ACS, was recently shown to stabilize the enzyme, leading to increased ACS activity and ACC content in wounded tomato tissue [12], [15].

Type 2 proteins are recruited by ETHYLENE-OVERPRODUCING1 (ETO1) and the ETO1-like EOL1 and EOL2 proteins for ubiquitin-dependent proteolysis [8], [9], [16], [17]. Ethylene overproduction in etiolated *eto1* seedlings is caused by decreased proteolytic turnover of type 2 ACS isozymes. The dominant *eto2* and *eto3* mutations, which alter C-terminal amino acid sequences of ACS5 and ACS9 required for recognition by ETO1 and EOL proteins, also cause ethylene overproduction in etiolated seedlings. Each of these mutations stabilizes the ACS^eto^ protein product by preventing its interaction with ETO1 [8], [9], [16], [18]. The CDPK target motif of type 2 ACS proteins can be phosphorylated, but the role of phosphorylation in regulating ACS accumulation has not yet been established [9], [11].

Although phosphorylation by CDPK and MAPK kinases has been linked to stabilization of ACS isozymes, ‘partner’ phosphatases acting on ACS isozymes have not been identified. The PPM-type protein phosphatase AP2C1 negatively regulates MPK6, and overexpression of AP2C1 compromises wound-induced ethylene production and disease resistance [19]. Okadaic acid and calyculin, inhibitors of protein phosphatase 1 (PP1) and PP2A, stabilize LeACS2 in tomato, and okadaic acid treatment causes the accumulation of LeACS2 phosphorylated at the CDPK site [12].

The heterotrimeric serine/threonine protein phosphatase PP2A was implicated in regulation of ethylene synthesis when the PP2A-deficient *rcn1* mutant was shown to overproduce ethylene [20]. Because ethylene is a potent inhibitor of cell expansion in dark-grown seedlings, ethylene overproduction results in a characteristic short hypocotyl phenotype in *rcn1* plants [20]–[22]. The *RCN1* gene encodes one of three regulatory/scaffolding A subunits of Arabidopsis PP2A, and the pleiotropic *rcn1* mutant phenotype results from a significantly reduced level of PP2A activity in *rcn1* plants [23], [24]. The experiments described here were designed to test the hypothesis that sustained C-terminal phosphorylation of ACS isozyme[s] due to decreased PP2A activity might account for ethylene overproduction in *rcn1* plants. Our results show that PP2A negatively regulates the activity and accumulation of type 1 ACS isozymes. PP2A directly interacts with the ACS6 protein, and PP2A complexes dephosphorylate a carboxy-terminal ACS6 phosphopeptide in vitro. Dephosphorylation requires the PP2A complexes that contain the RCN1 scaffolding subunit. Elicitor-mediated activation of MPK6, which up-regulates ethylene synthesis via type 1 ACS isozymes, has a reduced effect in the *rcn1* mutant background, consistent with the model that the baseline accumulation of phosphorylated type 1 ACS proteins is increased in *rcn1* plants. Genetic and molecular data also show that PP2A positively regulates the abundance of type 2 ACS proteins, revealing an unexpected role for PP2A in promoting accumulation of type 2 ACS isozymes. Our findings provide new insight into the finely tuned and phosphorylation-dependent regulation of ethylene synthesis.

## Results

### PP2A Inhibition Increases ACS Activity

**Figure 2:**
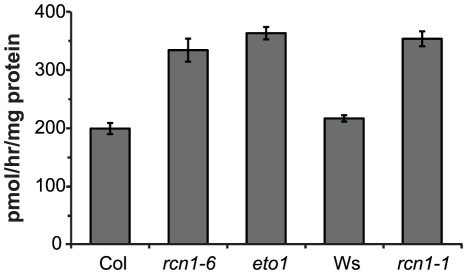
ACS enzymatic activity is increased in *rcn1* mutant seedlings. Total seedling proteins were extracted from etiolated wild-type and mutant plants five days post-germination. ACS enzymatic activities were assayed in triplicate (see Materials and Methods).

Ethylene biosynthesis is enhanced in dark-grown *rcn1* seedlings [20], [22]. Because ACC synthesis is generally the rate-limiting step for ethylene production during vegetative growth, we asked whether ACS activity was increased in *rcn1* mutant seedlings. We found that *rcn1* mutants in both the Columbia (Col) and Wassilewskija (Ws) genetic backgrounds exhibited increased ACS enzymatic activities in dark-grown seedlings (Figure 2). In the Col genetic background, *rcn1-6* and *eto1* seedlings showed similar ACS activity levels. These data suggest that ethylene overproduction and reduced hypocotyl lengths in *rcn1* mutant seedlings result at least in part from increased ACS enzymatic activity.

### PP2A-Mediated Regulation of Ethylene Production Requires Type 1 ACS Isozymes

**Figure 3:**
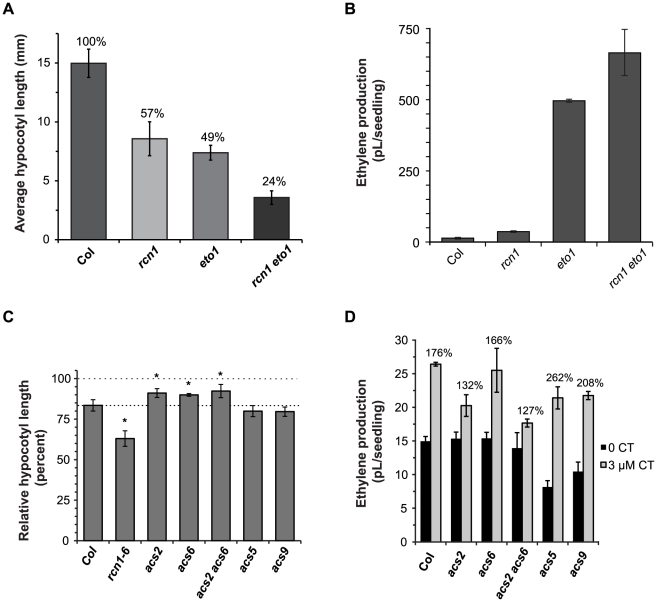
PP2A-dependent ethylene overproduction is *ETO1*-independent and requires type 1 isozymes. (A) The hypocotyl elongation defects of the *rcn1-1* and *eto1-1* single mutants are additive in the *rcn1 eto1* double mutant. Hypocotyl lengths of wild-type and mutant seedlings were measured after 5 days growth in the dark. Values shown represent average lengths; error bars show standard deviation (n>25). Shading differences indicate statistically significant differences in hypocotyl length (p<0.001). (B) The ethylene overproduction defects of the *rcn1-1* and *eto1-1* single mutants are additive in the *rcn1 eto1* double mutant. Wild-type and mutant seedlings were grown in sealed vials and ethylene levels were measured after 5 days growth in the dark. Values shown represent the average ethylene volume released per seedling. Error bars represent standard deviation (n≥4). (C) The cantharidin responsiveness of hypocotyl growth is reduced in plants carrying *acs2* and *acs6* mutations. Wild-type and mutant seedlings were grown in the presence or absence of 3 µM cantharidin, and hypocotyl lengths were measured after 5 days growth in the dark. Values shown represent average hypocotyl lengths of CT-grown plants as a percentage of the control length for that genotype; error bars show standard error (see Materials and Methods). Asterisks indicate a statistically significant difference in cantharidin response (p<0.05). (D) Cantharidin-induced ethylene synthesis is reduced in *acs2* and *acs2 acs6* mutants. Wild-type and mutant seedlings were grown in sealed vials in the presence or absence of 3 µM cantharidin and ethylene levels were measured as described above; the percent increase induced by cantharidin treatment is shown for each genotype. Error bars represent standard deviation (n = 3).

The *eto1* mutation causes ethylene overproduction by stabilizing type 2 ACS isozymes [8], [9], [16], [17]. In both *rcn1* and *eto1* mutants, ethylene overproduction reduces hypocotyl elongation in dark-grown seedlings, causing a short hypocotyl phenotype. To determine whether PP2A-mediated regulation of ethylene synthesis requires the ETO1 protein, we assayed hypocotyl elongation in *rcn1 eto1* double mutant seedlings. As expected, hypocotyl lengths in both single mutants were approximately one-half of those exhibited by the wild-type parents (Figure 3A). Seedlings carrying both the *rcn1* and *eto1* mutations exhibited an extreme reduction in hypocotyl length, corresponding to 25% of the wild-type parent. Double mutant seedlings also exhibited a significant increase in ethylene production above the level observed in either single mutant parent (p<0.001; Figure 3B). These data indicate that the *rcn1* and *eto1* defects in regulation of ethylene synthesis are additive, suggesting independent modes of action for PP2A and ETO1. Although the ethylene overproduction defect of *eto1* seedlings is more severe than that of *rcn1* seedlings, the hypocotyl lengths of the two mutants are similar in our assays (Figure 3B versus 3A). However, both the site (or source tissue) and timing of ethylene overproduction will affect the overall length of hypocotyls, and the *rcn1* and *eto1* mutants may differ significantly in these characteristics.

To determine whether PP2A-mediated regulation of ethylene synthesis targets particular ACS isozymes, we asked whether *acs* loss-of-function mutations affect the short hypocotyl phenotype caused by PP2A inhibition. Wild-type seedlings treated with the phosphatase inhibitor cantharidin (CT) exhibit a characteristic inhibition of hypocotyl elongation similar to that observed in untreated *rcn1* seedlings [21], [23], [25]. Hypocotyl elongation in the type 1 *acs* single mutants *acs2* and *acs6* and in the *acs2 acs6* double mutant showed decreased cantharidin response (Figure 3C). These data suggest that the effect of cantharidin on hypocotyl elongation is dependent on type 1 ACS isozymes. Similarly, ACS enzymatic activity in *acs2 acs6* double mutant seedlings was insensitive to cantharidin treatment, while normal seedlings treated with cantharidin exhibited increased ACS activity relative to untreated controls (data not shown). Interestingly, *acs5* and *acs9* loss-of-function mutants showed slightly increased cantharidin response (Figure 3C), indicating that the type 2 isozymes ACS5 and ACS9 are not required for cantharidin-mediated inhibition of hypocotyl elongation.

We also asked whether *acs* loss-of-function mutations affect ethylene overproduction in the presence of cantharidin. Wild-type seedlings treated with cantharidin exhibited a 76% increase in ethylene production (Figure 3D). While cantharidin-treated *acs6* seedlings exhibited a similar increase in ethylene synthesis, both *acs2* and *acs2 acs6* double mutant seedlings showed a reduced response to cantharidin treatment. Unlike the *acs2* and *acs6* mutants, *acs5* and *acs9* mutant seedlings exhibited a reduced baseline level of ethylene production. However, both type 2 *acs* mutants also showed a greater stimulation of ethylene synthesis in the presence of cantharidin (Figure 3D). Both the decreased basal ethylene synthesis and the increased response to cantharidin were more pronounced in the *acs5 acs9* double mutant, which exhibited a three-fold increase in ethylene production when grown in the presence of cantharidin (Figure S1). As in the hypocotyl elongation experiment described above, our data suggest that phosphatase inhibition acts through type 1 ACS isozymes to increase ethylene synthesis, while type 2 isozymes are not required for this effect. In addition, the data suggest that PP2A inhibition may have opposing effects on the activities of type 1 versus type 2 isozymes. This hypothesis is discussed in more detail below.

### Ethylene Synthesis in *rcn1* Plants Is Not Stimulated by Elicitor Treatment

**Figure 4:**
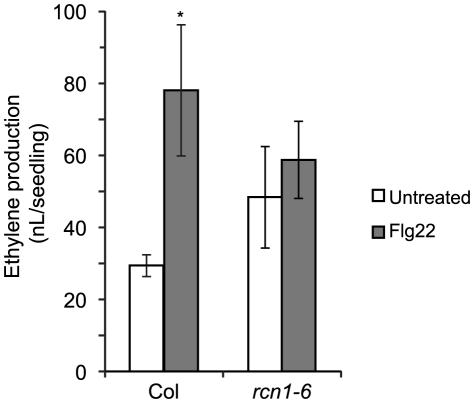
Flg22-induced ethylene production is reduced in *rcn1* seedlings. Wild-type and mutant seedlings were grown for 12 days in the light, and ethylene produced during a four-hour Flg22 treatment was measured (see Materials and Methods). Each value represents the average volume of ethylene released per seedling (n = 3). Error bars indicate standard deviation. Flg22 treatment induces a significant increase (*) only in wild-type Col seedlings (p<0.05).

Phosphorylation-mediated stabilization of type 1 ACS isozymes can be induced by treatment with the bacterial elicitor Flg22 [10], [13]. Because both the *rcn1* mutation and cantharidin treatment decrease PP2A activity levels, we expect that PP2A substrates will be hyperphosphorylated both in *rcn1* plants and in cantharidin-treated wild-type plants. We reasoned that the stabilizing effect of Flg22 treatment might be lessened if steady-state ACS phosphorylation levels are increased in the *rcn1* mutant. We measured Flg22-induced ethylene synthesis in wild-type (Col) and *rcn1-6* mutant seedlings, and found that *rcn1-6* seedlings indeed showed a reduced response to Flg22 treatment (Figure 4). While ethylene production rose by more than 2.5-fold (265%) in wild-type Col seedlings, the elicitor-induced increase in *rcn1-6* mutant plants (25%) was not statistically significant. Analysis of variance (ANOVA) shows that the difference between Flg22-treated wild-type (Col) seedlings and *rcn1-6* seedlings (Flg22-treated or untreated) also was not statistically significant. Flg22-induced ethylene synthesis in *rcn1-6* plants was not rescued by a higher Flg22 dose; treatment with 200 nM Flg22 resulted in a 262% increase in ethylene production in the wild type, and less than a 30% increase in *rcn1-6* plants (data not shown). The attenuated effect of Flg22 treatment in *rcn1-6* seedlings is consistent with the hypothesis that type 1 ACS isozymes are more phosphorylated and more stable when PP2A activity is reduced.

### PP2A Inhibition Stabilizes ACS6 Protein

**Figure 5:**
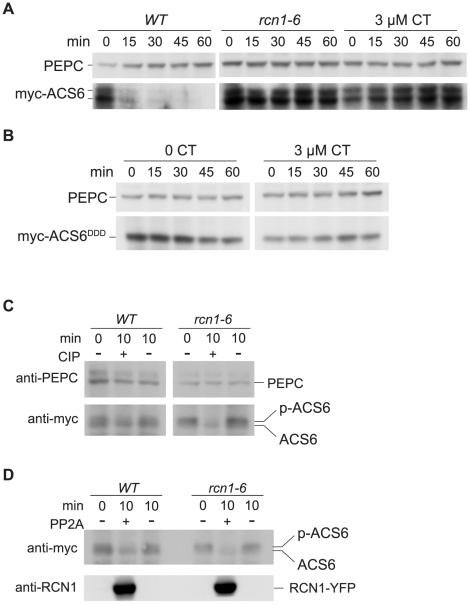
PP2A inhibition stabilizes ACS6 protein. Wild-type and *rcn1-6* seedlings expressing a wild-type *myc-ACS6* transgene (A) or a stabilized *myc-ACS6^DDD^* transgene (B) were grown in the absence or presence of 3 µM cantharidin (CT), and ACS6 turnover was assayed by immunoblotting extracts harvested at time points after addition of the protein synthesis inhibitor cycloheximide (see Materials and Methods). Immunoblots were probed with anti-myc to detect myc-ACS6 turnover and anti-PEPC as a loading control. (C) Extracts from wild-type and *rcn1-6* seedlings expressing myc-ACS6 were subjected to calf intestinal alkaline phosphatase (CIP) treatment (+) or incubated in buffer without CIP (−) for ten minutes. Samples were separated by SDS-PAGE to resolve the phosphorylated (P-ACS6) and dephosphorylated (ACS6) myc-ACS6 bands (see Materials and Methods). Immunoblots were probed with anti-PEPC (upper panel) as a loading control and with anti-myc (lower panel) to detect myc-ACS6. Migration of myc-ACS6 in treated samples can be compared to myc-ACS6 in untreated extracts (0 min) that were immediately boiled in SDS loading buffer. (D) Extracts from wild-type and *rcn1-6* seedlings expressing the wild-type myc-ACS6 were subjected to treatment with PP2A complexes (PP2A) immunoprecipitated from plants expressing RCN1-YFP (+), or incubated in buffer alone (−) for 10 minutes. Samples were processed as described for CIP-treated extracts (panel C above). Immunoblots were probed with anti-myc (upper panel) to detect myc-ACS6 and with anti-RCN1 (lower panel) to detect the RCN1-YFP fusion protein in PP2A-treated samples.

To directly assess the effect of PP2A inhibition on ACS6 protein turnover, we assayed the stability of an epitope-tagged ACS6 protein [10] in wild-type and *rcn1* mutant seedlings, and in wild-type plants grown in the presence of cantharidin. As expected, myc-ACS6 was rapidly turned over in dark-grown seedlings (Figure 5A). The *rcn1* mutation retarded the turnover of myc-ACS6, as did cantharidin treatment (Figure 5A). In contrast, the phosphomimic allele myc-ACS6^DDD^, which exhibits enhanced and phosphorylation-independent stability [13], was equally stable in wild-type seedlings grown in the absence or presence cantharidin (Figure 5B). Thus, the wild-type ACS6 protein is stabilized by phosphatase inhibition, while the phosphomimic ACS6^DDD^ protein is immune to this effect.

Immunoblots probed with anti-myc antibody revealed two poorly resolved bands near the molecular weight predicted for the myc-ACS6 protein. Extracts from *rcn1* mutants and from cantharidin-treated wild-type plants showed enhanced accumulation of the upper band (Figure 5A, 5C and 5D). To determine whether the upper and lower bands corresponded to phosphorylated and unphosphorylated myc-ACS6, we isolated protein extracts from *myc-ACS6*-expressing wild-type and *rcn1* plants and treated aliquots with alkaline phosphatase (Figure 5C) or with PP2A complexes immunoprecipitated from plants expressing an RCN1-YFP fusion protein (Figure 5D). Treatment with either CIP or PP2A resolved the upper and lower bands into a single species running at the position of the lower band (Figure 5C and 5D). These results are consistent with the hypothesis that both the *rcn1* lesion and phosphatase inhibition result in the accumulation of a more stable phosphorylated ACS6 species in vivo.

### ACS6 Protein Interacts with PP2A

**Figure 6:**
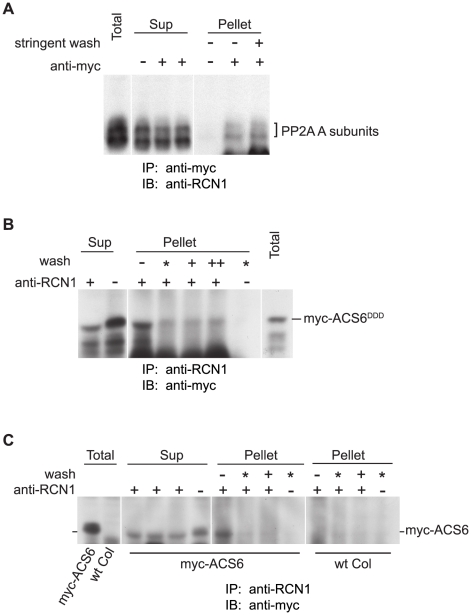
PP2A interacts with ACS6 in reciprocal co-immunoprecipitations. Protein extracts from MG132-treated seedlings expressing myc-ACS6^DDD^ (A, B) or wild-type myc-ACS6 (C) were subjected to immunoprecipitation (IP) with anti-myc (A) or anti-RCN1 (B, C) antibodies followed by immunoblotting (IB) with the antibodies indicated. Immunoprecipitates (Pellet fractions) were isolated without a wash (−), or were washed with a detergent-free buffer (*), with buffer containing 0.2% Tween-20 (+), or with buffer containing 0.2% Tween-20 plus 0.1% Triton X-100 (++). Samples of the supernatant fractions (Sup) were also analyzed to test for depletion of the putative interactor. Control immunoprecipitates (without anti-myc or anti-RCN1 antibodies) were washed with detergent-free buffer. Because the signal for wild-type myc-ACS6 was very weak in anti-RCN1 immunoprecipitations (C), extracts from wild-type Col plants lacking a *myc-ACS6* transgene were used as a control for background signals.

We asked whether the ACS6 protein interacts with PP2A complexes in planta. To address this question, we used a reciprocal co-immunoprecipitation approach. First we used anti-myc antibodies to immunoprecipitate ACS6 from protein extracts isolated from plants expressing myc-ACS6^DDD^ (Figure 6A). We probed these immunoprecipitates for regulatory A subunits of PP2A using anti-RCN1 antibodies that recognize all three A subunit isoforms [23], [26]. Regulatory A subunits were detected in the pellet fraction even after a detergent wash, suggesting a stable interaction between ACS6^DDD^ and PP2A. When anti-RCN1 antibodies were used for the reciprocal co-immunoprecipitation, immunoblotting revealed the presence of the myc-ACS6^DDD^ protein in the immunoprecipitates, and the ACS6 signal was maintained through a stringent detergent wash (Figure 6B). A weak but reproducible ACS6 signal was observed in anti-RCN1 immunoprecipitates isolated from plants expressing wild-type myc-ACS6; this signal was reduced after a gentle washing treatment, and was nearly undetectable after a stringent wash (Figure 6C). These co-immunoprecipitation data suggest that PP2A directly interacts with ACS6, and that interaction with the low-abundance wild-type isoform is unstable, while the interaction with the stabilized and more abundant ACS6^DDD^ protein is more robust. While the ACS6^DDD^ protein is an imperfect proxy for the phosphorylated wild-type ACS6 protein in this experiment, PP2A interaction may require motifs outside the MAPK phosphorylation site that are unaffected by the DDD substitution. For instance, in the case of PP2A-mediated dephosphorylation of the brassinosteroid-responsive transcription factor BRASSINAZOLE-RESISTANT1 (BZR1), a distinct binding motif is required for the PP2A interaction that leads to dephosphorylation of multiple sites; some or all of these sites lie outside the binding domain [27].

### PP2A Dephosphorylates an ACS6 Peptide Substrate *In Vitro*

**Figure 7:**
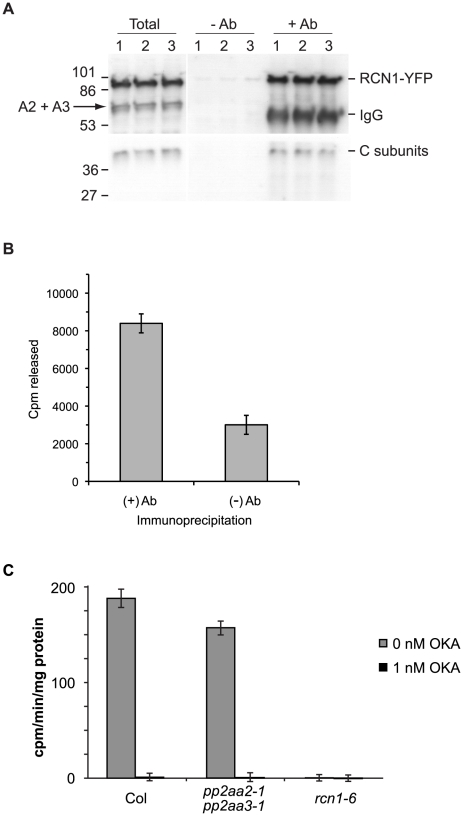
RCN1-containing PP2A complexes dephosphorylate the ACS6 C-terminal phosphopeptide. PP2A immunocomplexes were isolated and assayed for peptide dephosphorylating activity (A, B). Total protein extracts were prepared from three independent samples of dark-grown seedlings expressing an RCN1-YFP fusion protein [21]. Each extract was divided and immunocomplexes were isolated using anti-GFP antibodies (+Ab) or Protein A agarose beads alone (−Ab). Fractions of the total extracts (Total) and the immunoprecipitated pellets (−Ab and +Ab) were subjected to SDS-PAGE and immunoblotting (A) using anti-RCN1 (upper panel) and anti-C subunit (lower panel) antisera. Anti-RCN1 antibodies detect both the RCN1-YFP fusion protein and the PP2AA2 and PP2AA3 (A2+A3) proteins. (B) The (+) Ab and (−) Ab immunocomplexes were assayed for phosphatase activity against a ^33^P-labeled ACS6 phosphopeptide (see Materials and Methods). Values shown represent the average dephosphorylating activities present in the immunocomplexes. Error bars show standard error. Average dephosphorylation activities of (+) Ab and (−) Ab pellets are significantly different (p<0.05). (C) Crude extracts from wild-type and from *rcn1-6* and *pp2aa2-1 pp2aa3-1* mutant seedlings were assayed for ^33^P-labeled ACS6 phosphopeptide phosphatase activity in the presence or absence of 1 nM okadaic acid (OKA), which specifically inhibits PP2A (see Materials and Methods). Values shown represent the average peptide dephosphorylation activities. Error bars show standard deviation.

To determine whether PP2A acts directly on ACS6, we asked whether immunoprecipitated PP2A complexes could dephosphorylate an ACS6 phosphosubstrate. Because both native and recombinant ACS6 proteins are very unstable, we used a synthetic C-terminal peptide in our dephosphorylation assays. A C-terminal ACS6 30mer containing the three MPK target motifs was phosphorylated with recombinant MKK4^DD^ and MPK6 [13] and then used as a substrate for immunoprecipitated PP2A complexes isolated from transgenic plants expressing a YFP-tagged RCN1 (Figure 7A). PP2A immunocomplexes showed dephosphorylation activity against the ACS6 C-terminal peptide (Figure 7B). When the phosphatase inhibitor okadaic acid was added to the dephosphorylation reactions, activity dropped to background levels (data not shown). In combination with our finding that ACS6 and PP2A interact physically (Figure 6), these data suggest that ACS6 is a bona fide PP2A substrate in etiolated seedlings.

To determine whether a specific population of PP2A complexes may dephosphorylate ACS6, we tested the activities of crude lysates extracted from plants carrying mutations in genes encoding the three scaffolding A subunits of PP2A. Plants carrying the *rcn1* mutation maintain expression of the PP2AA2 and PP2AA3 scaffolds, while plants carrying the *pp2aa2-1 pp2aa3-1* double mutant combination express only the RCN1 scaffold [26]. Okadaic acid-sensitive ACS6 dephosphorylating activity was observed in extracts from wild-type and *pp2aa2-1 pp2aa3-1* seedlings, but not in extracts from *rcn1* plants (Figure 7C). In replicate experiments, extracts from *pp2aa2-1 pp2aa3-1* mutants showed 78±7% of wild-type activity levels, while *rcn1* mutants showed only background (0±1%) levels of activity (n = 4). These data indicate that RCN1-containing PP2A complexes are required for dephosphorylation of the ACS6 C-terminus. In contrast to the results obtained with this ACS6 peptide substrate, *rcn1* mutant plants exhibit approximately a two-fold decrease in ‘bulk’ PP2A activity (measured against the model substrate myelin basic protein; [22], [23]), while *pp2aa2-1 pp2aa3-1* plants exhibit 89±5% of wild-type bulk activity (J. Blakeslee, J. Heath and A. DeLong, unpublished). Thus our data indicate a specific requirement for the RCN1 scaffold in ACS6 dephosphorylation. These data are consistent with the specificity we observe in regulation of hypocotyl elongation; in contrast to the characteristic short hypocotyl phenotype of *rcn1* mutant plants (58.6±4.1% of wild-type), *pp2aa2-1 pp2aaa3-1* seedlings exhibit nearly normal hypocotyl lengths (96.7±3.6% of wild-type).

### PP2A Inhibition Reduces ACS5 Protein Accumulation

**Figure 8:**
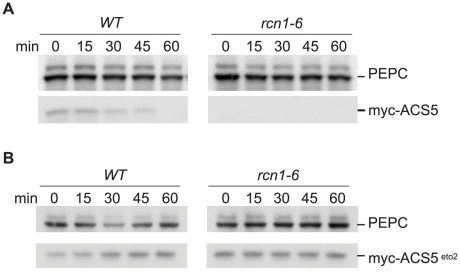
PP2A inhibition decreases ACS5 accumulation. Wild-type and *rcn1-6* mutant seedlings carrying Dexamethasone-inducible wild-type *myc-ACS5* (A) or *myc-ACS5^eto2^* (B) transgenes were grown on media containing 25 nM Dexamethasone and extracts were harvested at time points after addition of the protein synthesis inhibitor cycloheximide. Immunoblots were probed with anti-myc to detect myc-ACS5 proteins and with anti-PEPC as a loading control.

We observed a strikingly different effect of PP2A inhibition on ACS5 protein levels. As expected, normal seedlings exhibited rapid turnover of a myc-tagged ACS5 protein (Figure 8A). In *rcn1* seedlings, myc-ACS5 accumulation was dramatically reduced (Figure 8A). Direct comparisons of myc-ACS5 abundance in serial extract dilutions indicate that myc-ACS5 accumulation is reduced at least 25-fold in *rcn1* seedlings (see Figure S2B). We observed a similar decrease in myc-ACS5 accumulation when wild-type myc-ACS5 plants were treated with cantharidin (see Figure S2D). Although the reduced baseline accumulation of ACS5 in this experiment precludes accurate measurements of turnover under conditions of phosphatase inhibition, ACS5 protein does not appear to be stabilized by the *rcn1* mutation or by cantharidin treatment, but rather appears to be de-stabilized. These experiments employed an inducible myc-ACS5 construct [8] and reduced myc-ACS5 accumulation was apparent across a wide range of induction levels (25 to 200 nM Dexamethasone; see Figure S2A). At higher levels of induction, accumulation of the myc-ACS5 protein was more easily detected in *rcn1* mutant seedlings. Although turnover in wild-type seedlings was impeded at this expression level, myc-ACS5 was clearly unstable in the *rcn1* mutant plants (Figure S2C). Strikingly, accumulation and turnover of the stabilized myc-ACS5^eto2^ protein product was not affected by the *rcn1* mutation (Figure 8B). This result argues that the decreased accumulation of wild-type myc-ACS5 results from ACS5 protein instability, rather than any effect on the Dex-inducibility of transgene expression in *rcn1* plants. It also demonstrates that PP2A-dependent regulation of ACS5 accumulation requires the C-terminal sequences that are recognized by ETO1.

These observations are consistent with the increased cantharidin responsiveness of ethylene production and hypocotyl elongation we observed in *acs5* and *acs9* mutant plants (Figure 3C and 3D); in type 2 *acs* mutants, baseline ACS activity levels are reduced and the relative effect of stabilizing type 1 isozymes is exaggerated. Our data suggest that loss of PP2A activity simultaneously increases accumulation and activity of type 1 isozymes while reducing accumulation and activity of type 2 isozymes. Because normal baseline ACS activity levels are very low in dark-grown seedlings, stabilization of a single isozyme type has an obvious effect on ethylene production, even when other isozymes are destabilized. In effect, the increase in ethylene production due to stabilization of type 1 isozymes masks the destabilization of type 2 enzymes in hypocotyl elongation and ethylene biosynthesis experiments, and in the *eto1* epistasis experiment.

## Discussion

**Figure 9:**
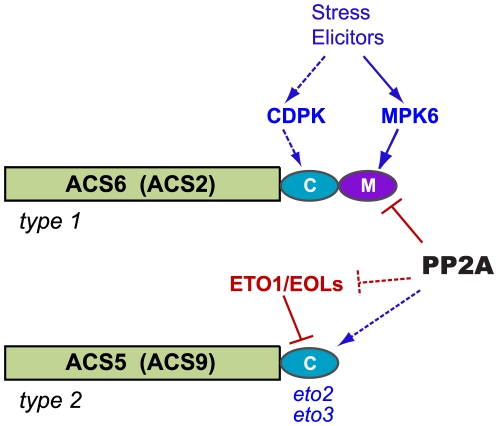
Model for PP2A-mediated regulation of ACS isozyme stability. Our results suggest that RCN1-containing PP2A destabilizes ACS6 through direct dephosphorylation of the C-terminus. This dephosphorylation opposes stabilizing C-terminal phosphorylation catalyzed by MPK6 (and possibly CDPK). Conversely, PP2A has a stabilizing effect on ACS5. Stabilization requires the wild-type C-terminal ACS5 sequence, and could involve direct action on type 2 ACS isozymes and/or inhibition of ETO1/EOL protein function. These possible modes of action are represented by dotted lines. Our genetic data indicate that PP2A-mediated regulation of ACS2 and ACS9 parallels that observed for ACS6 and ACS5, respectively.

Low levels of ethylene biosynthesis are characteristic of etiolated *A. thaliana* seedlings, and previous work has identified protein turnover mechanisms that limit the accumulation of ACS isozymes. In conjunction with the tight regulation of *ACS* mRNA levels [28], [29], these mechanisms constitute a stringent control system that regulates ethylene production [8], [10], [12], [13], [16], [30]. MAPK-mediated phosphorylation antagonizes the turnover mechanism that controls the stability of type 1 ACS isozymes in seedlings [10], [13], [19]. Our data indicate that RCN1-containing PP2A complexes dephosphorylate and promote the turnover of type 1 ACS isozymes in etiolated seedlings, suggesting that PP2A-mediated protein dephosphorylation is an important counterbalance to MAPK action. Conversely, PP2A appears to positively regulate the accumulation of type 2 ACS isozymes. Thus the control systems for type 1 and type 2 isozymes are independently specialized, but both involve PP2A action (Figure 9).

Under natural conditions, down-regulation of ethylene synthesis is necessary to allow the rapid hypocotyl cell expansion that ensures the emergence of seedling shoot tissues from the soil. Ethylene overproduction in plants with reduced PP2A activity results in a characteristic short hypocotyl phenotype [22], [25]. Exploiting that phenotype in our genetic analysis, we found that the *ACS2* and *ACS6* genes are required, while the *ETO1*, *ACS5* and *ACS9* genes are dispensable, for increased ethylene synthesis under conditions of PP2A inhibition. Direct analysis of ethylene production in *acs* loss-of-function mutants also demonstrated the requirement for type 1 but not type 2 isozymes. ACS enzyme activity levels are increased by *rcn1* mutations and by cantharidin treatment. These results support a model in which PP2A inhibition allows accumulation of phosphorylated and stabilized type 1 ACS isozymes. Further support for this model comes from our analysis of elicitor-induced ethylene production in wild-type and *rcn1* mutant plants. Wild-type plants exhibit a dramatic increase in ethylene production after Flg22 treatment, while *rcn1* plants, in which baseline ethylene production is elevated above the wild-type level, show only a modest increase. Turnover of the wild-type ACS6 protein is retarded in *rcn1* mutant plants and in cantharidin-treated wild-type plants, while the stabilized ACS6^DDD^ protein shows little or no effect of cantharidin treatment. The RCN1 protein interacts with both wild-type ACS6 and with the stabilized ACS6^DDD^ protein; as might be predicted for a substrate interaction, binding to the wild-type ACS6 protein appears quite unstable. Immunoprecipitated PP2A complexes dephosphorylate a MAPK- phosphorylated ACS6 C-terminal peptide. Finally, analysis of A subunit mutants shows that the RCN1 scaffolding subunit is required for dephosphorylation of the ACS C-terminal peptide, while loss of the PP2AA2 and PP2AA3 scaffolds has only a modest effect on dephosphorylation. This specific requirement for RCN1-directed dephosphorylation in vitro is mirrored by RCN1-specific regulation of hypocotyl elongation in vivo. Together these data suggest that PP2A complexes containing the RCN1 regulatory subunit dephosphorylate type 1 ACS isozymes, and that increased phosphorylation and stabilization of these enzymes allows increased ethylene synthesis in *rcn1* seedlings.

Recent work in tomato fruit indicates that LeACS2, a type 1 ACS isozyme of tomato, is stabilized when phosphorylated on both the CDPK and MAPK phosphorylation target sites [12]. Treatment with a protein phosphatase inhibitor promotes the accumulation of LeACS2 that is phosphorylated at the CDPK target site, increasing ACS activity levels. (The effect of protein phosphatase inhibition on phosphorylation at the MAPK sites was not directly assayed in that work.) The effect of phosphorylation at the putative CDPK site of *A. thaliana* type 1 ACS isozymes has not yet been tested. However, substitution of phosphomimic residues in the MAPK site is sufficient to dramatically increase the stability and accumulation of ACS6, suggesting that CDPK-dependent phosphorylation is not limiting for ACS6 stability in seedlings.

The mechanism by which phosphorylation stabilizes ACS isozymes has not been clearly defined. The non-catalytic carboxy-terminal domain of ACS6 is sufficient to confer 26S-proteasome-dependent instability on GFP and luciferase reporters, and it has been suggested that this region acts as a flexible docking domain that extends from the catalytic core. The distribution of acidic and basic residues in this region influences the degree of stabilization observed in the phosphomimicking ACS6^DDD^ mutant [10], consistent with the idea that phosphorylation at both the CDPK and MAPK sites could contribute to type 1 isozyme stability. Phosphorylation at the CDPK target site in type 2 isozymes was postulated to affect interactions with the ETO1/EOL-containing E3 ubiquitin ligase complex, but non-phosphorylatable and phosphomimic alleles of *ACS5* show normal interactions with *ETO1* and its paralogs in yeast 2-hybrid assays [9], indicating that modification at this site is not sufficient to regulate this critical interaction. Binding of 14-3-3 proteins to ACS isozymes also has been detected [31] and may play a role in phosphorylation-dependent stabilization.

Unexpectedly, PP2A appears to play a positive role in regulating the accumulation of ACS5, a type 2 isozyme. Thus the net result of phosphatase inhibition on ACS activity levels in wild-type plants represents the sum of two different effects: increased accumulation of type 1 isozymes and decreased accumulation of type 2 isozymes. The *rcn1* defect dramatically reduced the accumulation of ACS5 in plants carrying an inducible transgene construct, indicating that PP2A affects some post-transcriptional mechanism required for ACS5 accumulation. Our data suggest that type 2 ACS proteins are less stable when PP2A activity is reduced, but it is unclear whether this mechanism involves direct action on type 2 isozymes or dephosphorylation of a component of the ETO1 complex (Figure 9). We have not yet determined whether ETO1 plays a role in PP2A-mediated ACS5 stabilization. Recent proteomic profiling has identified the ETO1-like EOL1 and EOL2 proteins as well as one representative of each ACS isozyme type (ACS6, 7, 8) as 14-3-3 omega-binding clients, suggesting that these proteins are phosphorylated in vivo [31]. Since type 3 isozymes do not possess a C-terminal phosphorylation motif, these data suggest that phosphorylation in the conserved catalytic domain of some isozymes also may contribute to ACS regulation. The apparent enhancement of ethylene overproduction in cantharidin-treated *acs5* and *acs9* loss-of-function mutants indicates that PP2A function affects the activity of type 2 isozymes under native expression conditions as well. Although only *ACS1* and *ACS9* were found to make statistically significant contributions to control of hypocotyl elongation in 3-day old etiolated seedlings [32], our analysis of ethylene production shows that both *ACS5* and *ACS9* play important roles in ethylene synthesis in 5-day old seedlings, with *ACS2* and *ACS6* contributing little, when PP2A activity levels are normal. For both isozyme classes, fine-tuning of the activity levels requires protein phosphorylation/dephosphorylation and involves RCN1-regulated PP2A function.

Interestingly, when *acs2 acs6* double mutants are treated with cantharidin, ethylene production is slightly increased (Figure 3D). If overall ethylene production in *acs2 acs6* double mutants were solely dependent on type 2 ACS isozymes, we would predict that phosphatase inhibition would decrease ethylene synthesis. Phosphorylation-dependent regulation of the poorly understood type 3 isozymes may contribute to the residual cantharidin-induced ethylene synthesis observed in *acs2 acs6* double mutants. Additionally, recent analysis of single, double and multiple *acs* mutants has demonstrated that there is a complex interplay between ACS isozymes [32], and it is possible that a compensatory mechanism is activated in *acs2 acs6* double mutants.

Earlier work suggests that *ACS* mRNA levels also are affected by reversible protein phosphorylation [33]. The data reported here for accumulation of ACS5 and ACS6 proteins are derived from transgenic lines that employ constitutive (*35S::myc-ACS6*) and glucocorticoid-inducible (*myc-ACS5*) promoter fusions, and thus reflect effects of PP2A function that are independent of native *ACS* mRNA levels. Moreover, preliminary analysis of *ACS* mRNA levels suggests that *ACS6* transcript levels are normal, while *ACS5* and *ACS9* transcript levels increase, in *rcn1* mutant plants (M. Soruco and A. DeLong, unpublished). Since our genetic analysis indicates that ethylene overproduction requires *ACS2* and *ACS6*, but not *ACS5* and *ACS9*, these results suggest that effects on mRNA accumulation do not account for the ethylene overproduction phenotype of *rcn1* mutant seedlings. Similarly, analysis of enhanced LeACS2 accumulation after phosphatase inhibitor treatment indicates that mRNA levels remain unchanged while protein stability is increased [12].

## Materials and Methods

### Plant Material

To generate dark-grown seedlings for physiological and biochemical analysis, *A. thaliana* seeds were surface-sterilized, suspended in 0.1% agar and stratified for 3 days at 4°C before plating. Seedlings were grown on 0.5× Murashige and Skoog (MS) salts with 1% sucrose and 1% agar. After sowing, seeds were given a 16-hour light treatment before transfer into the dark for germination and growth at 24°C on vertical plates. Hypocotyl elongation, ACS activity and protein accumulation phenotypes were scored 5 days post germination. For hypocotyl elongation assays, approximately 30 seeds were sown on each plate; at the conclusion of the growth period, plates were imaged on a flatbed scanner. Hypocotyl lengths were measured using Image J. Cantharidin responsiveness of hypocotyl elongation was assayed in four separate experiments; results shown in Figure 3 represent the aggregate values ± standard error.

The *rcn1-1* mutant [24] and the *rcn1-1* RCN1-YFP transgenic line [21] are in the Wassilewskija (Ws) genetic background. The *rcn1-6* mutant [21] and all other mutants and transgenic lines used in this work are in the Columbia (Col) genetic background. Transgenic 35S::myc-ACS6, 35S::myc-ACS6^DDD^ and the *acs2* and *acs6* mutant lines [10] as well as the *acs2 acs6* double mutant [14] were the kind gift of S. Zhang (University of Missouri, Columbia). In *acs* loss-of-function experiments, we also used the *acs5-3* (*cin5*) and *acs9-1* alleles [28], [30]. Crossing *rcn1-1* and *eto1-1* generated the *rcn1 eto1* double mutant in a mixed Ws/Col background. Double mutant F3 families were compared to the parental single mutants and to single mutant sibling families segregating out of the cross.

### ACS Activity Assays

For ACS enzymatic activity assays, etiolated seedlings were harvested and ground in liquid nitrogen, resuspended in ACS protein extraction buffer [13] and assayed for ACS activity as described previously [8]. After chemical conversion of ACC to ethylene, 750 µl of headspace was transferred to a new vial for analysis on a Voyager portable gas chromatograph (PhotoVac Inc.). All reactions were carried out in triplicate.

### Protein Turnover Assays

For transgenic 35S::myc-ACS6 lines, MG-132 pre-treatment was used to promote protein accumulation [10]. For transgenic myc-ACS5 lines, seedlings were grown in the presence of low concentrations of Dexamethasone as previously described [8]. At the beginning of each turnover assay, seedlings were washed 3 times in liquid MS medium for 5 minutes and resuspended in liquid MS containing 1 mM cycloheximide. Samples were harvested and flash-frozen in liquid nitrogen in the dark at the specified time points and stored at −80°C until ACS stability was analyzed via immunoblotting with anti-myc antibody.

### Immunoblotting and Immunoprecipitation

For immunoblotting experiments, seedlings were ground to a fine powder in liquid nitrogen and boiled for 10 minutes with 4× SDS loading buffer (240 mM Tris pH 6.8, 8% SDS, 40% glycerol, 0.04% bromophenol blue, 5% beta-mercaptoethanol). Extracts were centrifuged at 16,000× g for 15 minutes at 4°C and supernatants were harvested for immediate use or storage at −20°C. Extracts were separated by electrophoresis on a 10% SDS-polyacrylamide gel, then transferred to a PVDF membrane (Millipore). Detection of proteins was performed using monoclonal anti-myc 9E10 (Covance), antisera against phospho-enol pyruvate carboxylase (anti-PEPC, Rockland) or polyclonal anti-RCN1 antibodies [23] and standard chemiluminescence.

For immunoprecipitation of PP2A complexes, RCN1-YFP seedlings were grown in the dark for 5 days on MS plates. Seedlings were then harvested and ground in liquid nitrogen, thawed in co-IP buffer (50 mM Tris, pH 7.5, 100 mM NaCl, 0.3 M sucrose, 0.2% Triton X-100, 2 µg/ml aprotinin and leupeptin). Extracts were centrifuged for 15 minutes at 16,000× g at 4°C to pellet debris, and the protein concentration of the supernatant was adjusted to 1.67 mg/ml with ice-cold co-IP buffer. For each precipitation, 250 µg of protein extract was incubated with 200 µl Protein A agarose (Invitrogen) plus 50 µl of 1.5∶100 dilution of anti-GFP antibody (AbCam) for 1 hour at 4°C. Immunoprecipitates were harvested by centrifugation at 1,000× g for 3 minutes at 4°C and washed twice in ice-cold co-IP buffer. For peptide dephosphorylation experiments, immunoprecipitates were resuspended in 200 µl of PPAB and aliquots were added to dephosphorylation reactions. For co-immunoprecipitation of ACS6 and PP2A, myc-ACS6 and myc-ACS6^DDD^ seedlings were grown in the dark on MS plates for 4.5 days and then transferred to liquid MS media containing MG-132 for 16 hours to allow ACS protein accumulation [10]. Protein extracts were prepared and processed with anti-myc or anti-RCN1 antibodies as described above, and immunoprecipitates were eluted from the Protein A agarose with SDS loading buffer, boiled and analyzed by immunoblotting as described above.

### Phosphatase Treatment of myc-ACS6

Protein extracts (62.5 µg total protein) from MG-132 treated myc-ACS6 seedlings were treated for 10 minutes at 37° with a phosphatase or with extraction buffer (100 mM HEPES, pH 7.5, 5 mM EDTA, 5 mM EGTA, 1 mM PMSF, 2 mM benzamidine, 2 µg/ml aprotinin and leupeptin) alone. For CIP treatment, 5 units of alkaline phosphatase (CIP, New England Biolabs) were added. For PP2A treatment, anti-GFP antibodies were used to immunoprecipitate PP2A complexes from seedlings expressing the RCN1-YFP fusion as described above; 10 µl of PP2A immunocomplexes were added to the myc-ACS6 extract. After phosphatase treatment, samples were boiled in SDS loading buffer and the migration of myc-ACS6 was analyzed by immunoblotting.

### Peptide Dephosphorylation Assays

Recombinant His-tagged MKK4^DD^ and MPK6 were purified using Ni-NTA affinity chromatography [13] and used to phosphorylate 500 µg of a biotinylated ACS6 peptide comprising the 30 C-terminal amino acids of ACS6 (generous gift of S. Zhang, University of Missouri, Columbia) in a buffer containing 200 µM ATP plus 12 µCi γ-^33^-P-ATP. Radiolabeled peptide (15 pmol/well) was then bound to a streptavidin-coated 96-well plate (Thermo Scientific) and incubated with shaking at 4°C for 2 hours. Each well was washed 4 times for 5 minutes with PPAB (50 mM Tris-HCl pH 7.0, 0.1 mM EDTA, 5 mM DTT, 0.01% Brij-35). For immunocomplex assays, PP2A complexes were isolated from plants expressing an RCN1-YFP fusion protein [21] using either a polyclonal anti-GFP antibody (AbCam) or Protein A agarose alone. After a 60-minute immunoprecipitation and two stringent washes at 4°C, IP pellets were resuspended in PPAB and 20 µl aliquots were assayed for ACS6 dephosphorylation activity (4 replicate reactions per IP pellet). After 15 minutes at 30°C, reactions were stopped with 4× loading buffer and each supernatant was sampled for released counts. Immunoprecipitation fractions also were subjected to immunoblot analysis using anti-RCN1 and anti-C subunit antibodies to confirm isolation of PP2A immunocomplexes. For crude extract assays, dark-grown seedlings were ground in liquid nitrogen and resuspended in PPAB. Extracts were diluted to a protein concentration of 2.5 µg/ml in PPAB containing okadaic acid at final concentrations of 0, 1 or 1000 nM, and 50 µl aliquots were added to 96-well plates pre-bound with 0.25 µg phospho-ACS6 peptide. Triplicate reactions were incubated at 30°C for 15 minutes before termination by addition of 4× loading buffer. Each supernatant was sampled for released counts, and the background activity observed at 1000 nM OKA was subtracted from the average values obtained in the presence of 0 and 1 nM OKA.

### Ethylene Evolution Measurements

For analysis of the effect of cantharidin on ethylene biosynthesis, seedlings were grown on 3 ml MS medium containing 3 µM cantharidin or a DMSO vehicle control in 22-mL gas chromatography vials for 5 days at 23°C in the dark. For Flg22 induction, seedlings were grown in long days at 23°C in 22-mL gas chromatography vials containing 3 ml MS medium for 12 days. Flg22 peptide (final concentration 40 µM) was added at day 12 and the vials were immediately capped and further incubated 4 hrs at 23°C in the light. In both experiments, the accumulated ethylene was measured by gas chromatography as described previously [30]. Flg22 peptide was the kind gift of S. Zhang, University of Missouri, Columbia.

## Supporting Information

Figure S1 Increased cantharidin responsiveness in *acs5 acs9* ethylene production. Wild-type, *acs5* and *acs5 acs9* seedlings were grown in sealed vials in the presence or absence of 3 µM cantharidin and ethylene levels were measured after 5 days growth in the dark. Each value shown represents the average amount of ethylene released per seedling; the percent increase induced by cantharidin treatment is shown for each genotype. Error bars represent standard deviation (n = 3). Different letters indicate significant differences (p<0.05).
(0.41 MB EPS)

Figure S2 The *rcn1-6* mutation reduces myc-ACS5 stability and accumulation. (A) Wild-type and *rcn1-6* seedlings carrying a Dexamethasone-inducible wild-type *myc-ACS5* transgene were grown in the dark on medium containing Dexamethasone at the concentrations indicated (nM). Seedlings were harvested and the abundance of myc-ACS5 in total protein extracts was assayed by immunoblotting with anti-myc and with anti-PEPC (loading control). (B) Extracts from wild-type plants were serially diluted in five-fold steps and subjected to immunoblotting to allow comparison of the abundance of myc-ACS5 in extracts from wild-type and *rcn1-6* seedlings grown on 25 nM Dexamethasone. Blots were probed with anti-myc and with anti-PEPC. (C) Wild-type and *rcn1-6* seedlings carrying *myc-ACS5* were grown on medium containing 125 nM Dexamethasone and extracts were harvested for immunoblotting at time points after addition of the protein synthesis inhibitor cycloheximide. Blots were probed with anti-myc to detect myc-ACS5 turnover and with anti-PEPC as a loading control. The short exposure of the anti-myc blot allows visualization of myc-ACS5 from wild-type samples, while the longer exposure shows protein turnover in *rcn1-6* seedlings. (D) Seedlings carrying *myc-ACS5* were grown on medium containing 20 nM Dexamethasone in the absence (−) or presence (CT) of 3 µM cantharidin and extracts were harvested for immunoblotting at time points after addition of the protein synthesis inhibitor cycloheximide. Blots were probed with anti-myc to detect myc-ACS5 turnover and with anti-PEPC as a loading control.
(6.18 MB EPS)
