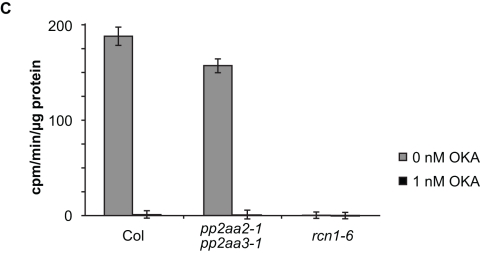# Correction: Protein Phosphatase 2A Controls Ethylene Biosynthesis by Differentially Regulating the Turnover of ACC Synthase Isoforms

**DOI:** 10.1371/annotation/b4fc15d6-b3ae-4fbb-8d88-b7d674a79697

**Published:** 2011-09-01

**Authors:** Kyle R. Skottke, Gyeong Mee Yoon, Joseph J. Kieber, Alison DeLong

In the y-axis of Figure 7, mg was mistakenly replaced by µg. Please view the correct figure here - 

**Figure:**